# Supplementary material for: Structural network alterations in focal and generalized epilepsy assessed in a worldwide ENIGMA study follow axes of epilepsy risk gene expression
Source: Nat Commun. 2022 Jul 27;13:4320. doi: 10.1038/s41467-022-31730-5 (PMC9329287; doi:10.1038/s41467-022-31730-5)
Supplement: Supplementary file 1 — Supplementary Information [file 41467_2022_31730_MOESM1_ESM.pdf]

STRUCTURAL NETWORK ALTERATIONS IN FOCAL AND GENERALIZED EPILEPSY ASSESSED IN A WORLDWIDE ENIGMA STUDY FOLLOW AXES OF EPILEPSY RISK GENE EXPRESSION

Supplementary Information

SUPPLEMENTARY TABLE 1. **Site-specific group demographics**. Site-specific demographic breakdown of patient-specific subcohorts, including age (in years), age at onset of epilepsy (in years), sex, side of seizure focus (TLE patients only), and mean duration of illness (in years). TLE=Temporal lobe epilepsy, IGE=idiopathic/genetic generalized epilepsy, HC=healthy controls.

**Site name**	**Case-controls subcohorts**		**Age** (mean±SD)	**Age at onset** (mean±SD)	**Sex** (male/female)	**Side of focus** (L/R)	**Duration of illness** (mean±SD)
**BERN**	TLE	*n*=18	31.28±9.09	–	9/9	10/8	–
	IGE	*n*=11	31.36±8.79	–	5/6	–	–
	HC	*n*=72	31.43±8.44	–	34/38	–	–
**BONN**	TLE	*n*=79	34.82±9.55	15.24±10.70	37/42	55/24	19.58±12.37
	IGE	*n*=0	–	–	–	–	–
	HC	*n*=57	34.09±9.69	–	30/27	–	–
**BRUSSELS**	TLE	*n*=13	35.85±5.27	12.50±10.60	5/8	10/3	23.25±13.21
	IGE	*n*=0	–	–	–	–	–
	HC	*n*=44	26.64±4.34	–	20/24	–	–
**CUBRIC**	TLE	*n*=0	–	–	–	–	–
	IGE	*n*=44	27.61±7.39	12.95±4.67	12/32	–	14.61±10.03
	HC	*n*=48	28.04±8.17	–	14/34	–	–
**EPICZ**	TLE	*n*=40	37.73±8.06	16.50±13.20	18/22	17/23	21.23±13.00
	IGE	*n*=0	–	–	–	–	–
	HC	*n*=96	35.58±8.80	–	47/49	–	–
**EPIGEN_3.0**	TLE	*n*=13	40.39±6.28	21.84±13.16	7/6	8/5	18.55±11.98
	IGE	*n*=0	–	–	–	–	–
	HC	*n*=64	33.11±8.00	–	35/29	–	–
**GREIFSWALD**	TLE	*n*=0	–	–	–	–	–
	IGE	*n*=25	32.56±7.85	19.44±9.73	12/13	–	13.12±9.96
	HC	*n*=96	25.76±5.00	–	36/60	–	–
**IDIBAPS-HCP**	TLE	*n*=46	35.02±8.43	15.22±11.28	21/25	14/32	18.97±10.35
	IGE	*n*=3	38.33±3.51	–	2/1	–	–
	HC	*n*=51	32.75±5.35	–	23/28	–	–
**KCL_CNS**	TLE	*n*=13	38.85±8.32	16.73±13.34	4/9	5/8	22.91±15.80
	IGE	*n*=31	29.94±8.42	12.00±5.73	11/20	–	17.94±8.93
	HC	*n*=97	30.82±7.39	–	45/52	–	–
**KUOPIO**	TLE	*n*=0	–	–	–	–	–
	IGE	*n*=35	27.86±8.34	17.46±9.93	13/22	–	10.46±10.67
	HC	*n*=67	25.16±1.55	–	34/33	–	–
**MNI**	TLE	*n*=79	33.10±8.82	17.09±10.08	34/45	44/35	16.01±10.72
	IGE	*n*=0	–	–	–	–	–
	HC	*n*=45	30.24±6.65	–	25/20	–	–
**NYU**	TLE	*n*=17	31.76±7.34	15.12±7.74	7/10	7/10	17.12±11.68
	IGE	*n*=35	32.43±9.34	14.23±5.25	19/16	–	18.38±9.86
	HC	*n*=108	28.51±8.88	–	52/56	–	–
**RMH**	TLE	*n*=25	34.80±9.22	23.24±12.72	15/10	16/9	12.03±12.51
	IGE	*n*=21	31.33±8.79	22.28±9.43	8/13	–	9.51±13.81
	HC	*n*=19	29.21±8.59	–	12/7	–	–

**Site name**	**Case-controls subcohorts**		**Age** (mean±SD)	**Age at onset** (mean±SD)	**Sex** (male/female)	**Side of focus** (L/R)	**Duration of illness** (mean±SD)
**UCSD**	TLE	*n*=17	33.24±8.42	14.87±12.60	8/9	10/7	19.53±13.53
	IGE	*n*=0	–	–	–	–	–
	HC	*n*=29	30.10±7.77	–	16/13	–	–
**UNAM**	TLE	*n*=17	30.76±9.50	14.53±12.73	7/10	9/8	16.18±9.54
	IGE	*n*=0	–	–	–	–	–
	HC	*n*=31	31.61±10.58	–	7/24	–	–
**UNICAMP**	TLE	*n*=161	40.83±7.64	11.49±9.70	70/91	91/70	29.65±11.55
	IGE	*n*=37	32.27±9.31	11.46±7.22	9/28	–	20.81±10.08
	HC	*n*=357	32.26±8.83	–	135/222	–	–
**UNIMORE**	TLE	*n*=0	–	–	–	–	–
	IGE	*n*=38	24.61±7.46	11.32±5.75	14/24	–	11.39±9.86
	HC	*n*=34	28.47±5.25	–	14/20	–	–
**XMU**	TLE	*n*=40	28.25±8.45	17.18±12.06	25/15	25/15	11.28±8.02
	IGE	*n*=11	31.00±10.45	18.56±16.49	7/4	–	12.00±10.23
	HC	*n*=13	31.54±6.99	–	9/4	–	–

SUPPLEMENTARY TABLE 2. List of disease-specific risk genes from previously published GWAS.

Disorder	Genes	Ref
All epilepsies	ADCY7, BCL11A, BRD7, CNEP1R1, COBLL1, CSRNP3, FANCL, GALNT3, HEATR3, SCN1A, SCN2A, SCN3A, SCN7A, SCN9A, TTC21B, VRK2	[1]
Childhood absence epilepsy	BCL11A, FANCL, GTDC1, VRK2	
Focal epilepsy	COBLL1, CSRNP3, GALNT3, SCN1A, SCN2A, SCN3A, SCN7A, SCN9A, TTC21B	[1]
Focal epilepsy (hippocampal sclerosis)	GJA1, GMPS, KCNAB1, PLCH1, SLC33A1	[1]
Generalized epilepsy	ATXN1, BCL11A, CCDC112, COBLL1, COPZ2, CSRNP3, FAM184A, FAM228A, FAM228B, FANCL, FKBP1B, GABRA2, GABRG1, GALNT3, GLS, GNPDA2, ITSN2, GRIK1, KCNN2, KLHL29, LRRC46, MFSD6, NAB1, NFE2L1, PCDH7, PFN4, PNPO, PTPRK, SCN1A, SCN2A, SCN3A, SCN7A, SCN9A, SCRN2, SKAP1, STAT1, STAT4, THEMIS, TP53I3, TRIM36, TTC21B, UBXN2A, VRK2	[1]
Juvenile myoclonic epilepsy	BCKDK, BCL7C, HSD3B7, ITGAM, ITGAX, KAT8, ORAI3, PYCARD, PYDC1, STX1B, STX4, ZNF629, ZNF646	[1]
Anti-epileptic drug targets	CA12, CA2, CA4, CACNA1B, CACNA1G, CACNA1H, CACNA1I, CACNA2D1, CHRNA2, CHRNA4, CHRNA7, GABRA1, GABRA2, GABRA3, GABRA4, GABRA5, GABRB1, GABRB2, GABRB3, GABRD, GABRE, GABRG1, GABRG2, GABRG3, GRIA1, GRIA2, GRIA3, GRIA4, GRIK1, GRIK2, GRIK3, GRIK4, GRIK5, GRIN2A, GRIN2B, GRIN2C, GRIN3A, PDGFRA, SCN1A, SCN2A, SCN3A, SCN7A, SCN8A, SCN9A	[2]
Monogenic epilepsy	ALDH7A1, ALG13, ARHGEF9, ARX, CACNA1A, CDKL5, CHD2, CHRNA2, CHRNA4, CHRNA7, CLN6, CNTNAP2, CSTB, CTSD, DEPDC5, DNAJC5, DNM1, EEF1A2, EPM2A, FOLR1, FOXG1, GABRA1, GABRB2, GABRB3, GABRG2, GAMT, GOSR2, GRIN2A, GRIN2B, HCN1, IQSEC2, KANSL1, KCNA2, KCNB1, KCNC1, KCNMA1, KCNQ2, KCNT1, MAGI2, MBD5, MECP2, MEF2C, NHLRC1, NR2F1, NRXN1, PCDH19, PIGO, PNPO, PRICKLE1, PRICKLE2, PRRT2, SCN1A, SCN1B, SCN2A, SCN8A, SCN9A, SLC25A22, SLC2A1, SLC35A2, SLC6A8, SLC9A6, STX1B, STXBP1, SYNGAP1, TBC1D24, TPP1, TSC1, WDR45, WWOX	[3], GeneDX (www.genedx.com)
Attention deficit/ hyperactivity disorder	B4GALT2, CCDC24, DPH2, DUSP6, FOXP2, IPO13, LINC00461, PCDH7, POC1B, PTPRF, SEMA6D, SORCS3, SPAG16, ST3GAL3, TMEM161B_AS1	[4]
Autism spectrum disorder	APOPT1, BAG5, CADPS, CKB, FEZF2, KCNN2, KIZ, KLC1, KMT2E, MACROD2, MROH5, NEGR1, NKX2_2, NUDT12, PINX1, POU3F2, PTBP2, SOX7, TRMT61A	[5]

Bipolar disorder	ADCY2, ADD3, ANK3, CACNA1C, FADS2, FSTL5, GRIN2A, HDAC5, LMAN2L, MRPS33, NCAN, PACS1, PC, PLEKHO1, POU3F2, RIMS1, RPS6KA2, SCN2A, SHANK2, SSBP2, STARD9, STK4, THSD7A, TRANK1, ZNF592	6
Major depressive disorder	ANKHD1, ANKS1B, AP3B1, APOPT1, ARHGEF25, ASCC3, ASIC2, ASTN2, ASXL3, ATP1A3, BAD, BAG5, BAZ2B, BCHE, BSN, BTN2A1, BTN3A2, BTN3A3, C16orf45, CABP1, CACNA1E, CACNA2D1, CAMKK2, CCS, CDH13, CDH22, CDH9, CDK14, CELF2, CELF4, CHD6, CKB, CNTN5, CNTNAP5, CRB1, CSMD1, CTNNA3, CTTNBP2, DCC, DENND1A, DENND1B, DRD2, ELAVL2, EMILIN3, EPHB2, ERBB4, ESRRG, ETFDH, EXT1, FADS1, FADS2, FAM120AOS, FAM172A, FANCL, FCF1, FHIT, FNIP2, GINM1, GPC5, GPC6, GRIK5, GRM5, GRM8, GTF2IRD1, HARS, HIST1H4L, HLA_DQB1, IGSF6, INPP4B, KDM3A, KIF15, KLC1, KYNU, LIN28B, LRFN5, LRP1B, LST1, MAP9, MED19, MEF2C, MEGF11, METTL9, MGAT4C, MICB, MIER1, MR1, MYBPC3, NEGR1, NICN1, NRG1, OLFM4, PAX6, PCDHA1, PCDHA5, PCLO, PLA2R1, PLCL1, POGZ, PPP6C, PRR16, PRSS16, PSEN2, PSORS1C1, PTPRS, RAB27B, RAB3B, RABEPK, RBFOX1, RBMS3, RHOBTB1, RSRC1, RTN1, SAMD5, SCAI, SDK1, SEMA6D, SERPING1, SF3B1, SGIP1, SHISA9, SLC4A9, SORBS3, SORCS3, SOX5, SOX6, SPPL3, TCTEX1D1, TENM2, TMEM258, TMEM42, TMEM67, TRAF3, TRMT10C, TRMT61A, TTC12, UBE2M, USP3, VRK2, YLPM1, ZDHHC21, ZDHHC5, ZFHX4, ZKSCAN8, ZMAT2, ZNF165, ZNF184, ZNF322, ZNF35, ZNF660, ZSCAN26, ZSCAN31, ZSCAN9	7
Migraine	ASTN2, DOCK4, FUT9, GJA1, GPR149, HEY2, HPSE2, ITPK1, JAG1, MPPED2, MRVI1, NOTCH4, NRP1, PHACTR1, PLCE1, PRDM16, SLC24A3, SPINK2, TSPAN2, WSCD1, YAP1, ZCCHC14	8
Schizophrenia	ABCB9, ACTR5, ADAMTSL3, ANP32E, APOPT1, ARHGAP1, ARL3, ASPHD1, ATP2A2, BAG5, C12orf65, C1orf54, C2orf69, CA14, CACNA1C, CACNA1I, CACNB2, CCDC39, CDK2AP1, CHADL, CHRM4, CHRNA3, CKB, CNKSR2, CNNM2, CNTN4, DGKZ, DNAJC19, DOC2A, DPYD, DRD2, EFHD1, ERCC4, ESAM, FAM57B, FANCL, FES, FUT9, FXR1, GATAD2A, GIGYF2, GNL3, GRM3, HAPLN4, HARBI1, HSPE1, INA, IREB2, KCNV1, KLC1, L3MBTL2, MAD1L1, MAN2A2, MAPK3, MDK, MPHOSPH9, MSANTD2, MSL2, NAGA, NCAN, NDUFA13, NDUFA6, NEK1, NEK4, NGEF, NISCH, NRGN, NT5DC2, OGFOD2, OTUD7B, PBRM1, PBX4, PCCB, PCGF6, PITPNM2, PJA1, PLCH2, PLCL1, PLEKHO1, PPP1R16B, PTPRF, R3HDM2, RILPL2, SBNO1, SEPT3, SF3B1, SGSM2, SHISA8, SHMT2, SLC32A1, SLC39A8, SLC45A1, SMIM4, SNAP91, SREBF2, SRR, STAB1, STAG1, STAT6, TAC3, TAF5, TMEM219, TRANK1, TRIM8, TRMT61A, TSNARE1, TYW5, VRK2, YPEL3, ZFYVE21	9

SUPPLEMENTARY TABLE 3. **Site-specific recruitment and ethics committees**. Locations and periods of participant recruitment, and details of research ethics committee approval at each site.

Site name	Location of recruitment	Period of recruitment	Research ethics committee
**BERN**	Inselspital Universitätsspital Bern, Switzerland	2009–2015	Ethics commission of the Canton of Bern. Signed consent forms were acquired to use images for research.
**BONN**	University Hospital Bonn, Germany	2006–2011	Medical Ethics Committee of the University of Bonn. Signed consent forms were acquired to use images for research.
**BRUSSELS**	Hospital Erasme, Universite ibre de Bruxelles, Brussels, Belgium	2005–2015	Ethics Commission of Erasme Hospital. Consent forms were acquired to use images for research.
**CUBRIC**	Cardiff University Brain Research Imaging Centre (CUBRIC), Cardiff, United Kingdom	2009–2015	Southmead Research Ethics Committee, Bristol, UK (ref: 08/H0102/12).
**EPICZ**	Epilepsy Centre Catanzaro (EPICZ), Italy	2010–2015	Research Ethics Committee of University “Magna Graecia” (ref: 2010/03/31).
**EPIGEN_3.0**	Epilepsy Genetics (EPIGEN) Dublin, St. James’s Hospital, Dublin, Ireland	2010–2014	SJH/TUH Joint Research Ethics Committee (JREC; ref: 2011/10/01).
**GREIFSWALD**	Department of Neurology, University Medicine Greifswald, Germany	2010–2015	University Medicine Greifswald's Ethics Committee. Signed consent forms were acquired to use images for research.
**IDIBAPS-HCP**	Institut D’Investigacions Biomèdiques August Pi I Sunyer research center, Hospital Clínic Barcelona (IDIBAPSHCB), Cataluña, Spain	2007–2015	The Ethics Committee of Hospital Clinic de Barcelona. Consent forms were acquired to use images for research.
**KCL_CNS**	King’s College London Centre for Neuroimaging Sciences (KCL_CNS), London, United Kingdom	2007–2012	KCL College Research Ethics Committees (ref: 08/H0808/157).
**KUOPIO**	Kuopio University Hospital, Finland	2010–2015	The Research Ethics Committee of the Northern Savo Hospital (ref: 128/13.02.00/2015).
**MNI**	Montreal Neurological Institute (MNI), McGill University, Montreal, Canada	2008–2015	The Ethics Committee of the Montreal Neurological Institute

Site name	Location of recruitment	Period of recruitment	Research ethics committee
			and Hospital (ref: NEU-08-001; BERA 2002/1).
**NYU**	New York University, New York, USA	2006–2015	New York University's Institutional Review Board (ref: s12-02038; 11224).
**RMH**	Royal Melbourne Hospital (RMH), Melbourne, Australia	2008–2013	The Royal Melbourne Hospital Human Research Ethics Committee (HREC; ref: QA2012044).
**UCSD**	University California San Diego (UCSD), California, USA	2010–2015	The institutional review board at UC San Diego (ref: 120297).
**UNAM**	Universidad Nacional Autónoma de México (UNAM), Campus Juriquilla, Querétaro, México	2013–2015	The Ethics Committee of the Neurobiology Institute of the Universidad Nacional Autónoma de México (ref: 019.H-RM).
**UNICAMP**	Universidade Estadual de Campinas (UNICAMP), Brazil	2010–2015	Comitê de Ética em Pesquisa da Universidade Estadual de Campinas (ref: CEP1158/2009).
**UNIMORE**	Università degli Studi di Modena (UNIMORE), Modena, Italy	2010–2015	The human ethics committee of the University of Modena and Reggio Emilia (ref: n. 80/10, n. 155/14).
**XMU**	Xiamen University (XMU), Xiamen, China	2012–2015	Xinjiang Medical University (XMU) Research Ethics Committee. Signed consent forms were acquired to use images for research.

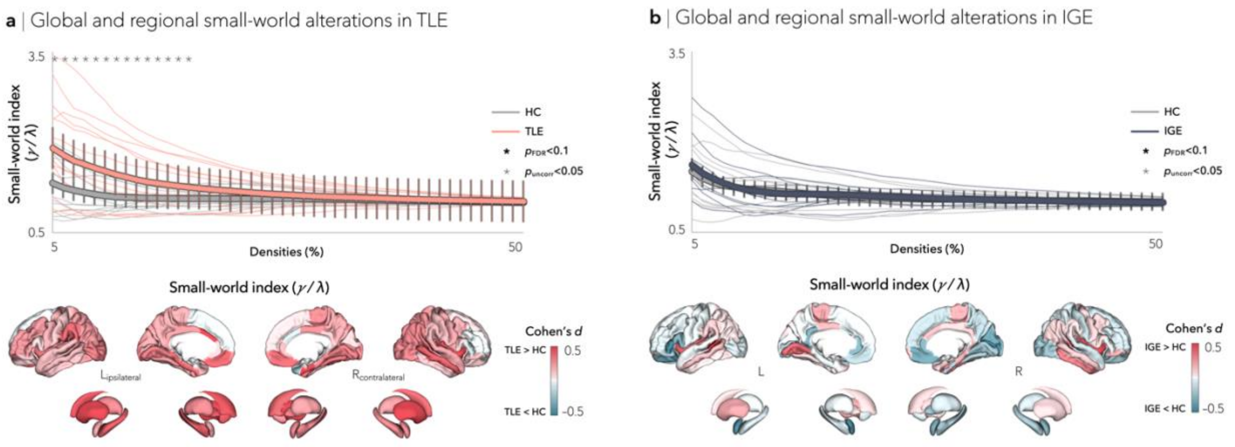

Supplementary Figure 1. **Small-world organization in TLE and IGE**. (**a**) Global (top) and regional (bottom) differences in small-world index between temporal lobe epilepsy (TLE) and healthy controls (HC) are plotted as a function of network density. Two-tailed student's *t*-tests comparing TLE patients to controls were performed at each density value; trends for increased small-worldness was observed in individuals with TLE (p_{uncorr}<0.05). Bold asterisks indicate p_{FDR}<0.1, semi-transparent asterisks indicate p_{uncorr}<0.05. Thin lines represent data from individual sites. Error bars indicate standard error of the mean. Cohen's *d* effect sizes pointed to overall increases in regional small-world index in TLE. (**b**) Global (top) and regional (bottom) differences in small-world index between idiopathic generalized epilepsy (IGE) and HC are plotted as a function of network density. Two-tailed student's *t*-tests comparing IGE patients to controls were performed at each density value; overall small-world organization was virtually identical in individuals with IGE and HC. Thin lines represent data from individual sites. Error bars indicate standard error of the mean. Cohen's *d* effect sizes pointed to slight increases and decreases in regional small-world index in IGE. *Abbreviations*: HC = healthy control, IGE = idiopathic generalized epilepsy, TLE = temporal lobe epilepsy, p_{FDR} = *p*-value adjusted for false discovery rate, p_{uncorr} = uncorrected *p*-value.

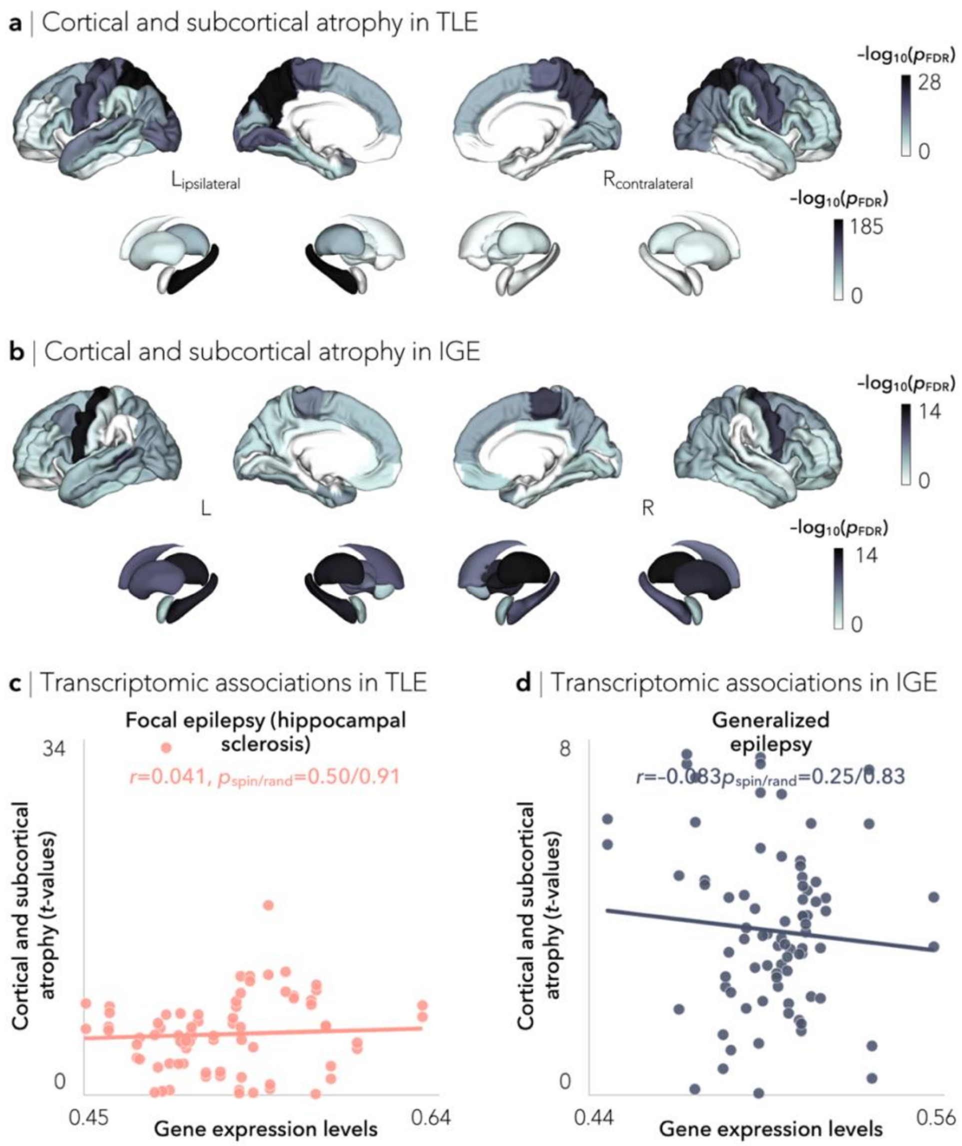

Supplementary Figure 2. **Associations between cortical/subcortical atrophy and epilepsy risk genes**. (**a**) Two-tailed surface-based linear models compared atrophy profiles in patients relative to controls, correcting for multiple comparisons using the false discovery rate (FDR) procedure. Cortical thickness and subcortical volume reductions in TLE, compared to healthy controls, spanned bilateral superior parietal (left/right $p_{FDR}=2.86\times10^{-29}/4.50\times10^{-27}$), precuneus (left/right $p_{FDR}=3.54\times10^{-29}/3.32\times10^{-22}$), precentral (left/right $p_{FDR}=3.95\times10^{-21}/3.12\times10^{-20}$), and paracentral (left/right $p_{FDR}=1.75\times10^{-19}/5.41\times10^{-18}$) cortices, as well as ipsilateral hippocampus ($p_{FDR}=2.32\times10^{-186}$) and thalamus ($p_{FDR}=1.25\times10^{-67}$). (**b**) Two-tailed surface-based linear models compared atrophy profiles in patients relative to controls, correcting for multiple comparisons using the false discovery rate (FDR) procedure. In contrast to TLE, grey matter cortical and subcortical atrophy in IGE, relative to controls, was more subtle and affected predominantly bilateral precentral cortices (left/right $p_{FDR}=2.94\times10^{-14}/7.75\times10^{-12}$) and thalamus (left/right $p_{FDR}=8.63\times10^{-14}/1.70\times10^{-14}$). Negative $\log_{10}$-transformed FDR-corrected *p*-values are shown. (**c**) Imaging-transcriptomics associations in TLE were computed across cortical and subcortical regions (n = 82) and statistically assessed using one-tailed non-parametric tests. Transcriptomic associations were significantly weaker when derived from regional atrophy patterns (as opposed to multivariate topological changes) in TLE (correlation with gene expression levels of hippocampal sclerosis: r=0.041, $p_{spin/rand}$=0.50/0.91). (**d**) Similarly, imaging-transcriptomics associations in IGE were computed across cortical and subcortical regions (n = 82) and statistically assessed using one-tailed non-parametric tests. Transcriptomic associations were also significantly weaker when derived from regional atrophy patterns in IGE (correlation with gene expression levels of generalized epilepsy: r=–0.083, $p_{spin/rand}$=0.25/0.83). *Abbreviations*: HC = healthy control, IGE = idiopathic generalized epilepsy, TLE = temporal lobe epilepsy, p_{FDR} = *p*-value adjusted for false discovery rate.

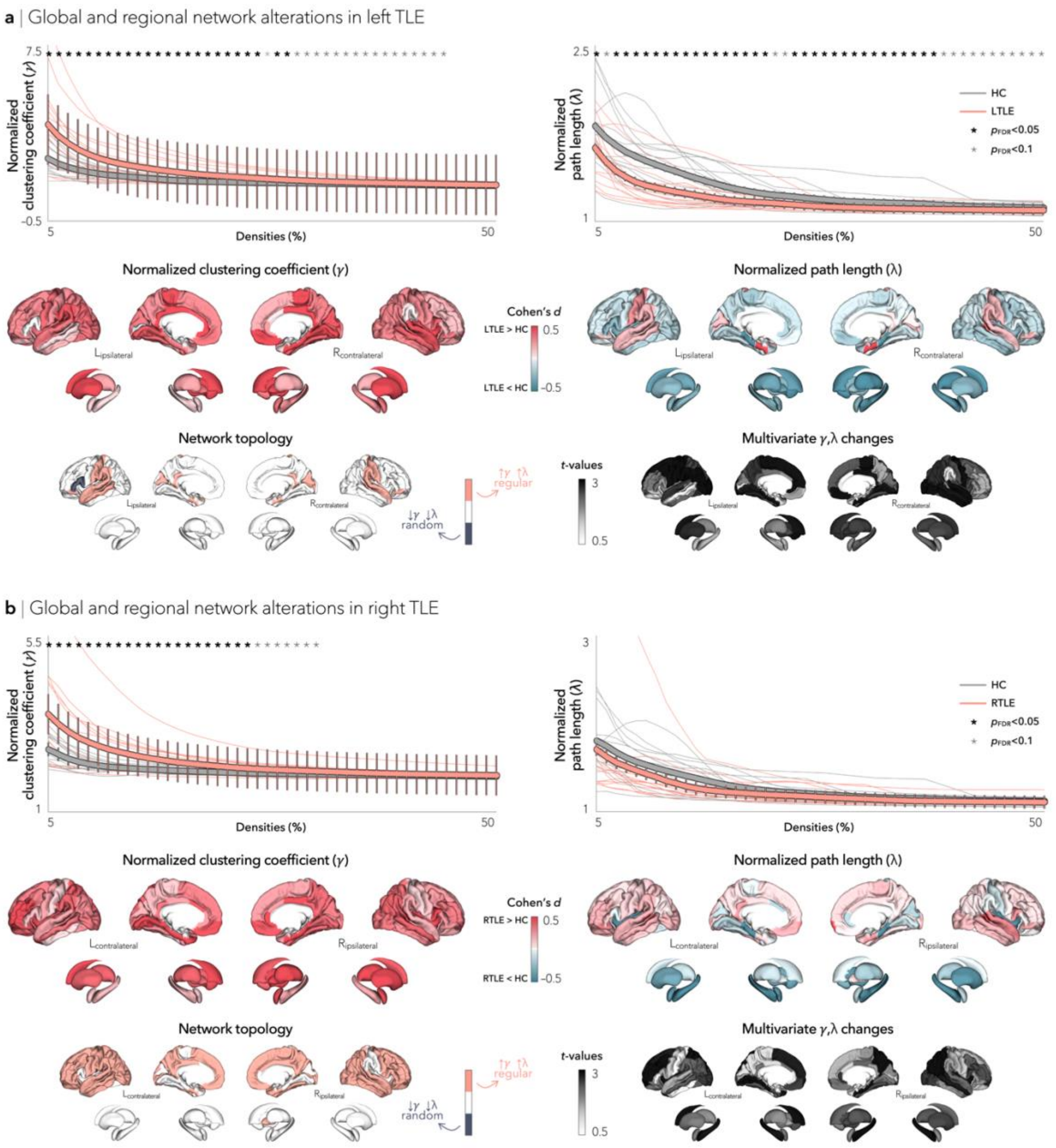

Supplementary Figure 3. **Structural covariance networks in left (LTLE) and right (RTLE) TLE**. (**a**) Global differences in clustering coefficient (*top left*) and path length (*top right*) between left LTLE and healthy controls (HC) are plotted as a function of network density. Increased small-worldness (increased clustering coefficient, decreased path length) was observed in individuals with left TLE. Student's *t*-tests comparing LTLE patients to controls were performed at each density value; bold asterisks indicate $p_{FDR}<0.05$, semi-transparent asterisks indicate $p_{FDR}<0.1$. Thin lines represent data from individual sites. Error bars indicate standard error of the mean. Multivariate topological differences in left TLE were primarily observed in bilateral fronto-temporal cortices, and revealed a regular network configuration (increased clustering and path length). (**b**) Global differences in clustering coefficient (*top left*) and path length (*top right*) between RTLE and HC are plotted as a function of network density. Increased small-worldness (increased clustering coefficient, decreased path length) was observed in individuals with right TLE. Student's *t*-tests comparing RTLE patients to controls were performed at each density value; bold asterisks indicate $p_{FDR}<0.05$, semi-transparent asterisks indicate $p_{FDR}<0.1$. Thin lines represent data from individual sites. Error bars indicate standard error of the mean. Multivariate topological changes in right TLE were primarily observed in bilateral fronto-temporal cortices and the hippocampus, and revealed a widespread regular network configuration. *Abbreviations*: HC = healthy control, LTLE = left temporal lobe epilepsy, RTLE = right temporal lobe epilepsy, p_{FDR} = *p*-value adjusted for false discovery rate.

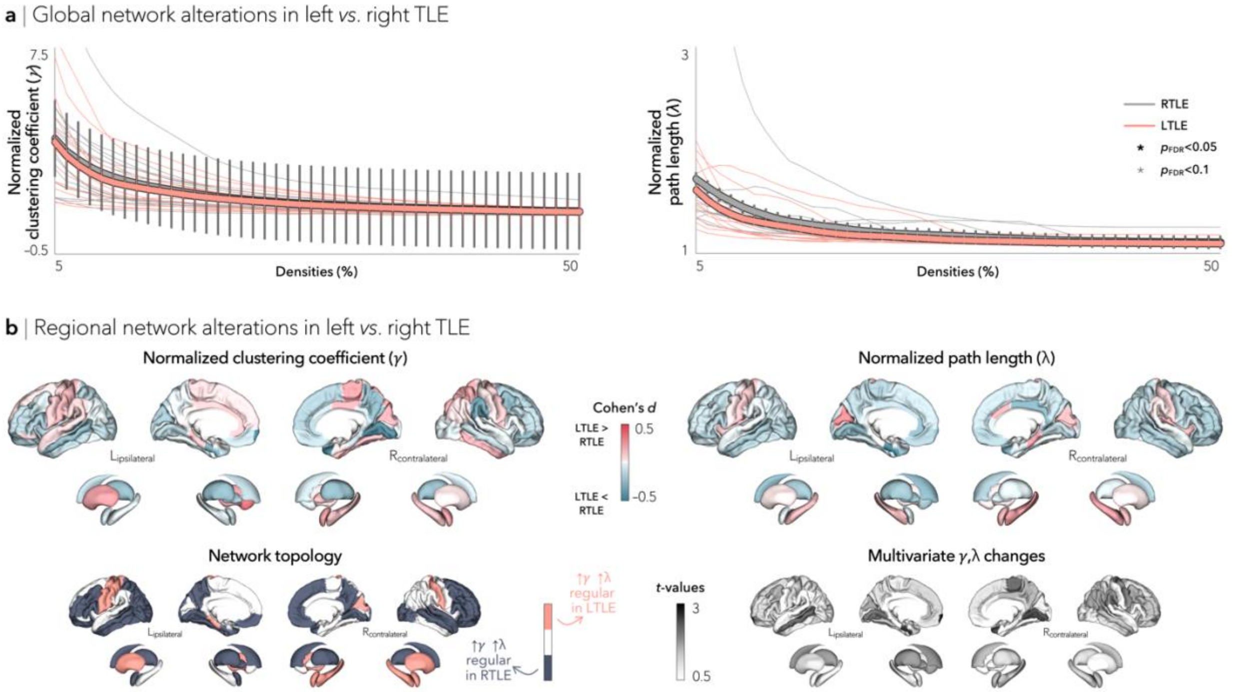

Supplementary Figure 4. **Structural covariance networks in left (LTLE) *vs*. right (RTLE) TLE**. (**a**) Global differences in clustering coefficient (*left*) and path length (*right*) between LTLE and RTLE are plotted as a function of network density. No significant difference was observed. Two-tailed student's *t*-tests were performed at each density value; bold asterisks indicate $p_{FDR}<0.05$, semi-transparent asterisks indicate $p_{FDR}<0.1$. Thin lines represent data from individual sites. Error bars indicate standard error of the mean. (**b**) Trends for multivariate topological changes in LTLE *vs*. RTLE were observed in ipsilateral middle frontal gyrus and enthorinal cortex as well as contralateral calcarine sulcus. Compared to the other subcohort, LTLE showed network regularization (increased clustering and path length) in sensorimotor cortices, whereas RTLE showed widespread network regularization. *Abbreviations*: LTLE = left temporal lobe epilepsy, RTLE = right temporal lobe epilepsy, p_{FDR} = *p*-value adjusted for false discovery rate.

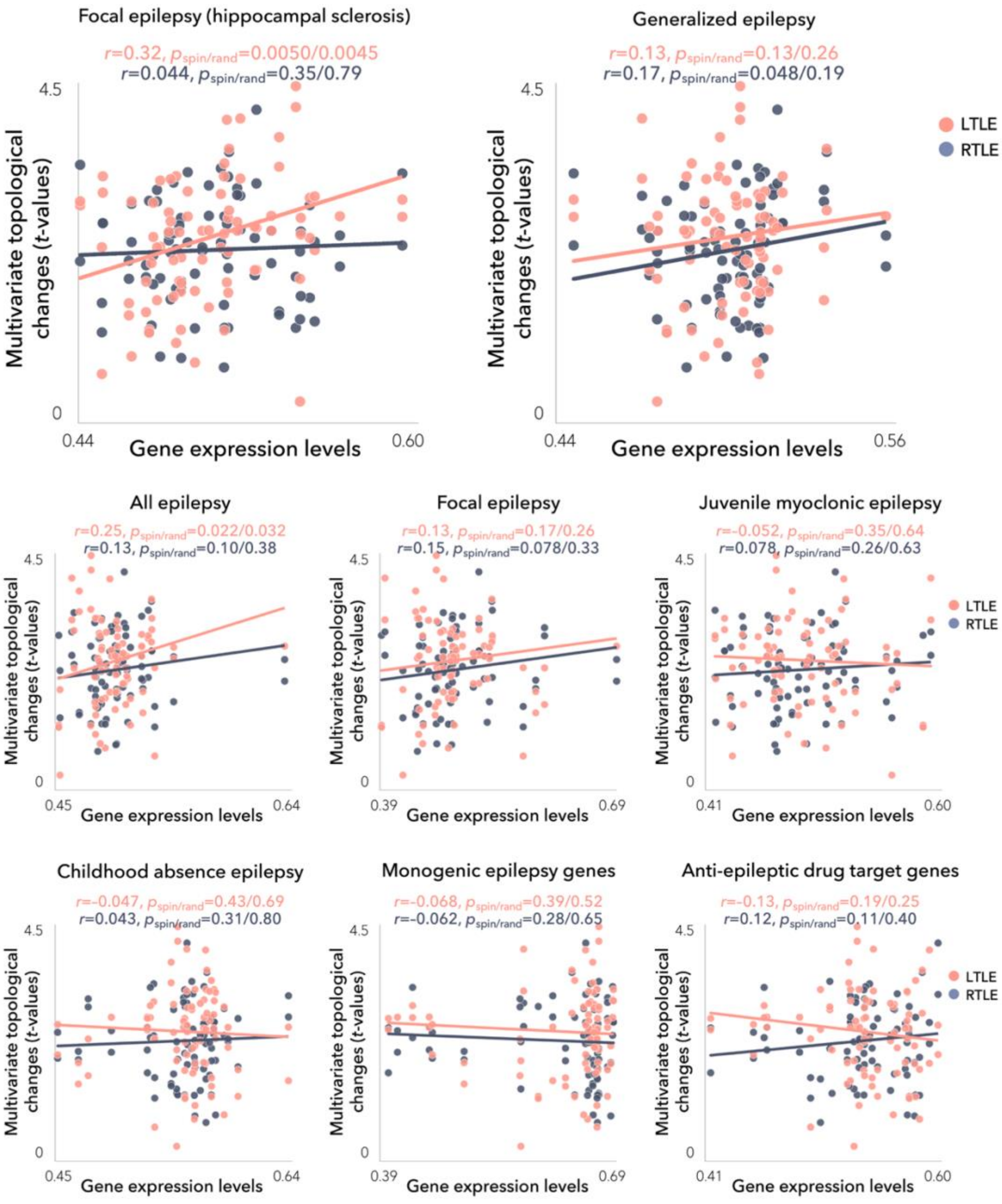

Supplementary Figure 5. **Relations between epilepsy gene expression and network topology**. Spatial correlations were performed between gene expression levels associated with (*i*) every epilepsy subtype (focal epilepsy with hippocampal sclerosis, generalized epilepsy, all epilepsy, focal epilepsy, juvenile myoclonic epilepsy, and childhood absence epilepsy), (*ii*) monogenic epilepsy, and (*iii*) anti-epileptic drug targets and the patterns of multivariate topological alterations in left (LTLE) and right (RTLE) TLE across cortical and subcortical regions (n = 82). Statistical significance was assessed using one-tailed, non-parametric tests. In left TLE, spatial associations between microarray data and multivariate topological changes were significant for expression levels of focal epilepsy with hippocampal sclerosis (r=0.32, $p_{spin/rand}$=0.0050/0.0045) and all epilepsy (r=0.25, $p_{spin/rand}$=0.0022/0.032). In right TLE, network associations only correlated with transcriptomic maps of generalized epilepsy (r=0.32, p_{spin}=0.048), but did not survive correction against a "random-gene" null distribution (p_{rand}=0.19). *Abbreviations*: LTLE = left temporal lobe epilepsy, RTLE = right temporal lobe epilepsy, p_{spin} = p-value corrected against a null distribution of effects using a spatial permutation model, p_{rand} = p-value corrected against a null distribution of effects using a "random-gene" permutation model.

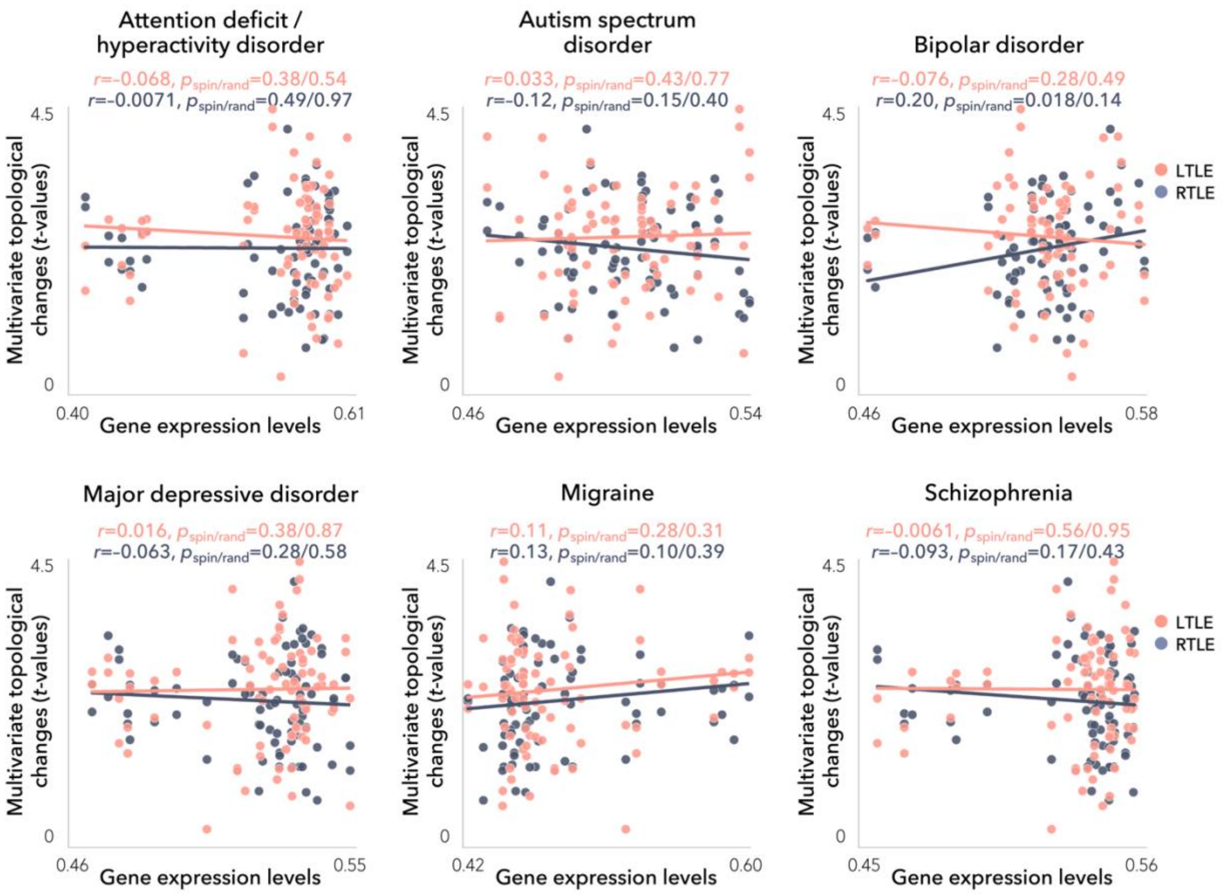

Supplementary Figure 6. **Relations between disease-related gene expression and network topology**. Spatial correlations were performed between gene expression levels associated with six common neuropsychiatric conditions and/or comorbities of epilepsy (attention deficit/hyperactivity disorder, autism spectrum disorder, bipolar disorder, major depressive disorder, migraine, and schizophrenia) and the patterns of multivariate topological alterations in left (LTLE) and right (RTLE) TLE across cortical and subcortical regions (n = 82). Statistical significance was assessed using one-tailed, non-parametric tests. In left TLE network associations did not correlate with any other epilepsy-related transcriptomic maps. In right TLE, network associations correlated with transcriptomic maps of bipolar disorder (r=0.20, p_{spin}=0.018), but did not survive correction against a "random-gene" null distribution (p_{rand}=0.14). *Abbreviations*: LTLE = left temporal lobe epilepsy, RTLE = right temporal lobe epilepsy, p_{spin} = p-value corrected against a null distribution of effects using a spatial permutation model, p_{rand} = p-value corrected against a null distribution of effects using a "random-gene" permutation model.

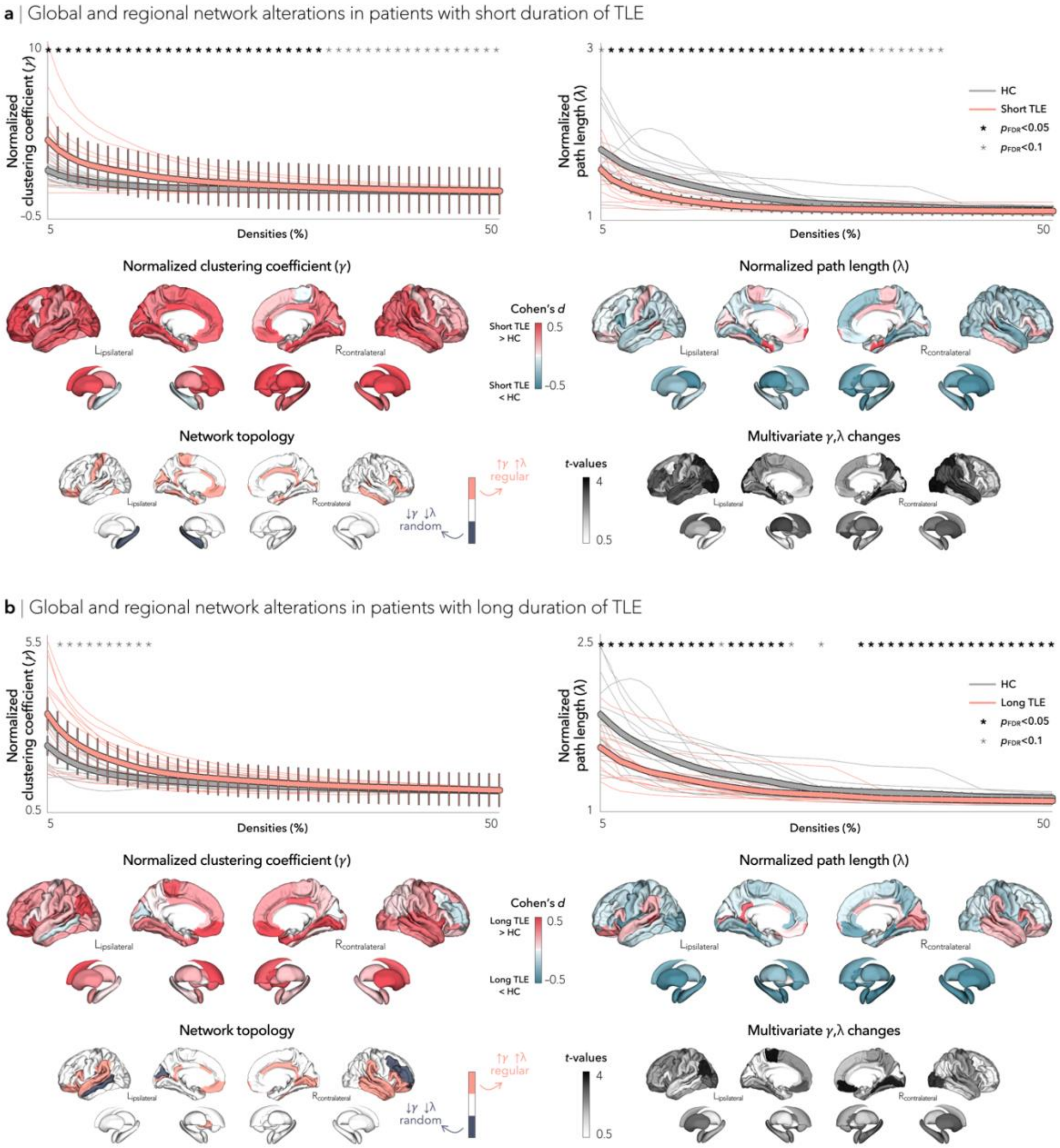

Supplementary Figure 7. **Structural covariance networks in patients with short and long TLE duration**. (**a**) Global differences in clustering coefficient (*top left*) and path length (*top right*) between Short TLE and healthy controls (HC) are plotted as a function of network density. Increased small-worldness (increased clustering coefficient, decreased path length) was observed in patients with short TLE duration. Two-tailed student's *t*-tests were performed at each density value; bold asterisks indicate $p_{FDR}<0.05$, semi-transparent asterisks indicate $p_{FDR}<0.1$. Thin lines represent data from individual sites. Error bars indicate standard error of the mean. Multivariate topological changes in patients with short TLE duration were widespread, affecting bilateral fronto-parietal and limbic cortices and the thalamus, and revealed a regular subnetwork configuration (increased clustering and path length). (**b**) Global differences in clustering coefficient (*top left*) and path length (*top right*) between Long TLE and healthy controls (HC) are plotted as a function of network density. Increased small-worldness (increased clustering coefficient, decreased path length) was observed in patients with long TLE duration. Two-tailed student's *t*-tests were performed at each density value; bold asterisks indicate $p_{FDR}<0.05$, semi-transparent asterisks indicate $p_{FDR}<0.1$. Thin lines represent data from individual sites. Error bars indicate standard error of the mean. Multivariate topological changes in patients with long TLE duration were primarily observed in fronto-limbic cortices and bilateral putamen, amygdala, and hippocampus, and revealed a regular subnetwork configuration (increased clustering and path length). *Abbreviations*: HC = healthy control, TLE = temporal lobe epilepsy, p_{FDR} = *p*-value adjusted for false discovery rate.

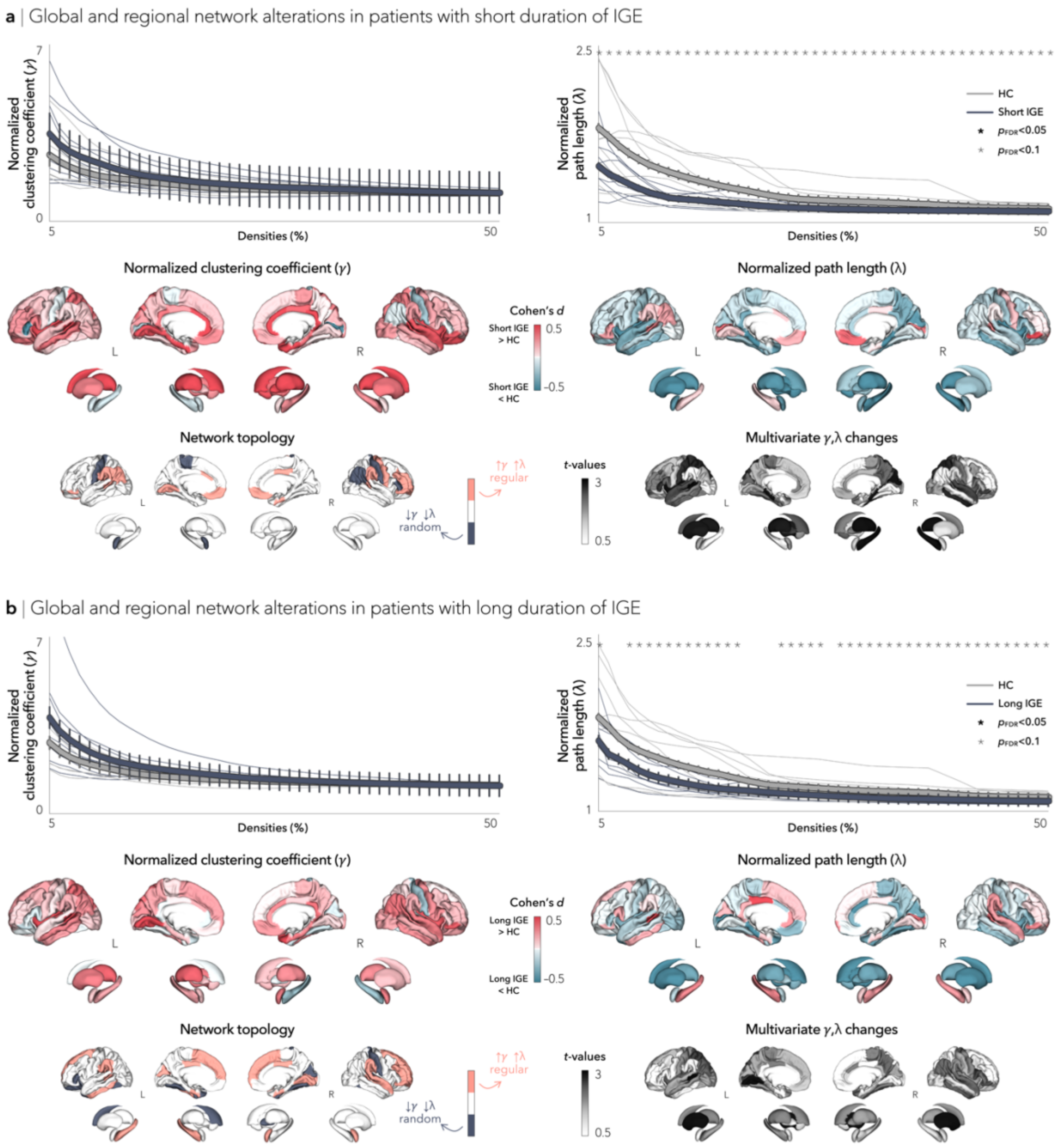

Supplementary Figure 8. **Structural covariance networks in patients with short and long IGE duration**. (**a**) Global differences in clustering coefficient (*top left*) and path length (*top right*) between Short IGE and healthy controls (HC) are plotted as a function of network density. Decreased path length was observed in patients with short IGE duration. Two-tailed student's *t*-tests were performed at each density value; bold asterisks indicate $p_{FDR}<0.05$, semi-transparent asterisks indicate $p_{FDR}<0.1$. Thin lines represent data from individual sites. Error bars indicate standard error of the mean. Multivariate topological changes in patients with short IGE duration were observed, primarily in bilateral fronto-central and temporal cortices, as well as bilateral thalamus and right hippocampus. Both random (decreased clustering and path length in centro-parietal regions) and regular (increased clustering and path length in orbitofrontal and parietal regions) network configurations were observed. (**b**) Global differences in clustering coefficient (*top left*) and path length (*top right*) between Long IGE and healthy controls (HC) are plotted as a function of network density. Decreased path length was observed in patients with long IGE duration. Two-tailed student's *t*-tests were performed at each density value; bold asterisks indicate $p_{FDR}<0.05$, semi-transparent asterisks indicate $p_{FDR}<0.1$. Thin lines represent data from individual sites. Error bars indicate standard error of the mean. Multivariate topological changes in patients with long IGE duration were primarily observed in bilateral fronto-temporo-parietal regions and bilateral putamen. Once again, both random (fronto-central cortices) and regular (fronto-temporo-parietal regions) network configurations were observed. *Abbreviations*: HC = healthy control, IGE = idiopathic generalized epilepsy, p_{FDR} = *p*-value adjusted for false discovery rate.

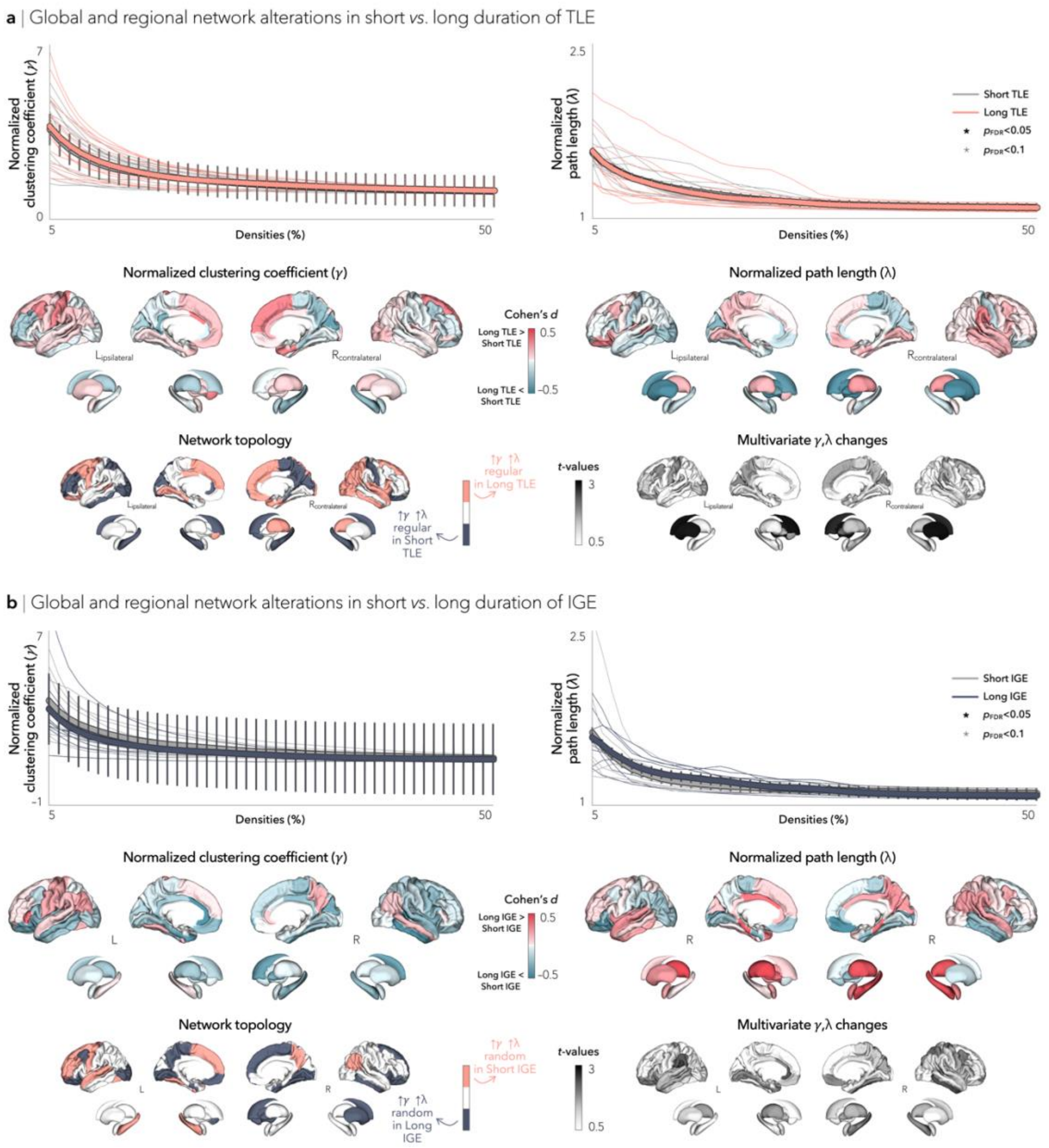

Supplementary Figure 9. **Structural covariance networks in short *vs.* long TLE/IGE duration**. (**a**) Global differences in clustering coefficient (*top left*) and path length (*top right*) between Short and Long TLE are plotted as a function of network density; no significant difference was observed in clustering coefficient or path length. Two-tailed student's *t*-tests were performed at each density value; bold asterisks indicate $p_{FDR}<0.05$, semi-transparent asterisks indicate $p_{FDR}<0.1$. Thin lines represent data from individual sites. Error bars indicate standard error of the mean. Marginal multivariate topological changes were observed between groups, primarily affecting bilateral caudate and ipsilateral putamen. Patients with shorter duration exhibited network regularization (increased clustering and path length) in bilateral fronto-parietal cortices and hippocampus, whereas patients with longer duration exhibited network regularization more broadly in fronto-temporo-parietal cortices. (**b**) Global differences in clustering coefficient (*top left*) and path length (*top right*) between Short and Long IGE are plotted as a function of network density; no significant difference was observed in clustering coefficient or path length. Two-tailed student's *t*-tests were performed at each density value; bold asterisks indicate $p_{FDR}<0.05$, semi-transparent asterisks indicate $p_{FDR}<0.1$. Thin lines represent data from individual sites. Error bars indicate standard error of the mean. Marginal multivariate topological changes were observed between groups, primarily affecting bilateral fronto-limbic cortices. Patients with shorter duration exhibited network randomization (decreased clustering and path length) in left fronto-temporal cortices and hippocampus and right parietal cortices, whereas patients with longer duration exhibited network randomization in left fronto-parieto-occipital cortices and right fronto-temporal cortices, caudate, and putamen. *Abbreviations*: IGE = idiopathic generalized epilepsy, p_{FDR} = *p*-value adjusted for false discovery rate.

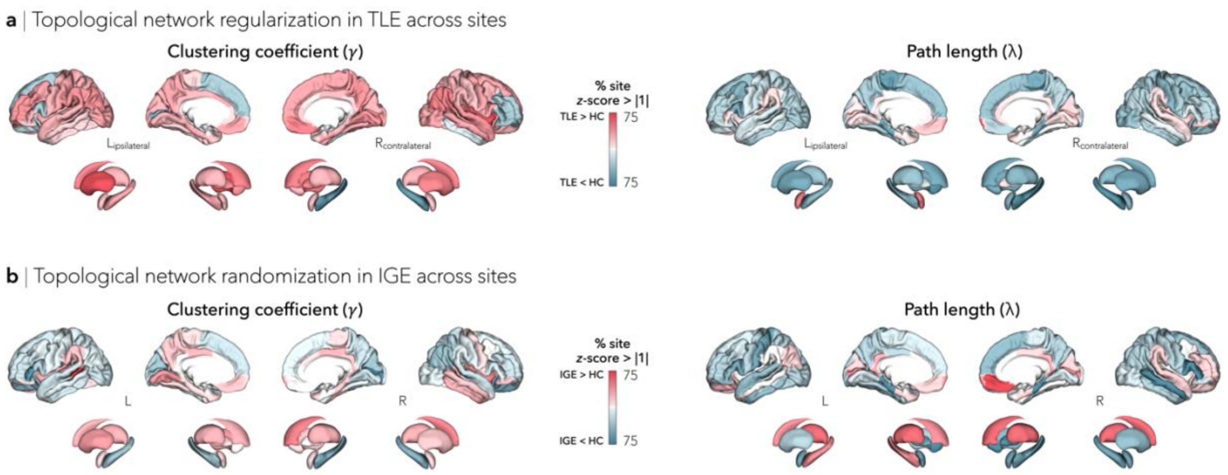

Supplementary Figure 10. **Site-specific structural covariance changes in TLE and IGE**. Structural covariance and graph theoretical analyses were repeated in each site independently and yielded virtually identical results. (**a**) In TLE, increased clustering and path length were observed in bilateral orbitofrontal, temporal, and angular cortices, as well as ipsilateral amygdala, revealing a regularized, "lattice-like," subnetwork. (**b**) In IGE, widespread decreased in clustering and path length were observed in fronto-temporo-parietal regions, suggesting a randomized network configuration. *Abbreviations*: HC = healthy control, IGE = idiopathic generalized epilepsy, TLE = temporal lobe epilepsy.

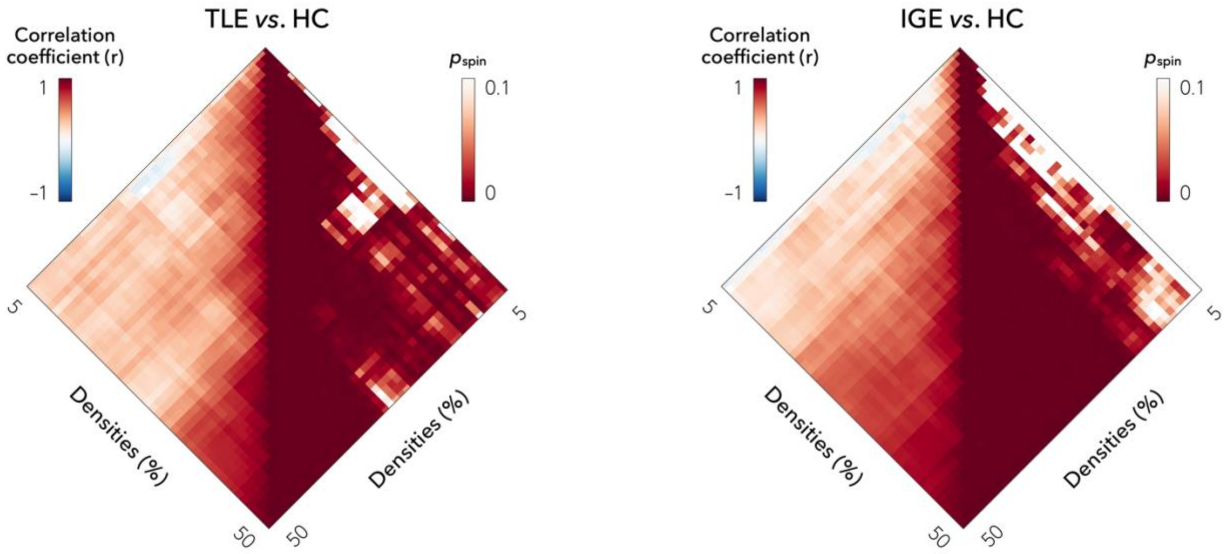

Supplementary Figure 11. **Stability of multivariate network findings across matrix thresholds**. Associations between multivariate topological changes (clustering and path length) across matrix thresholds. Statistical significance was assessed using one-tailed, non-parametric tests. *Abbreviations*: HC = healthy control, IGE = idiopathic generalized epilepsy, TLE = temporal lobe epilepsy, p_{spin} = *p*-value corrected against a null distribution of effects using a spatial permutation model.

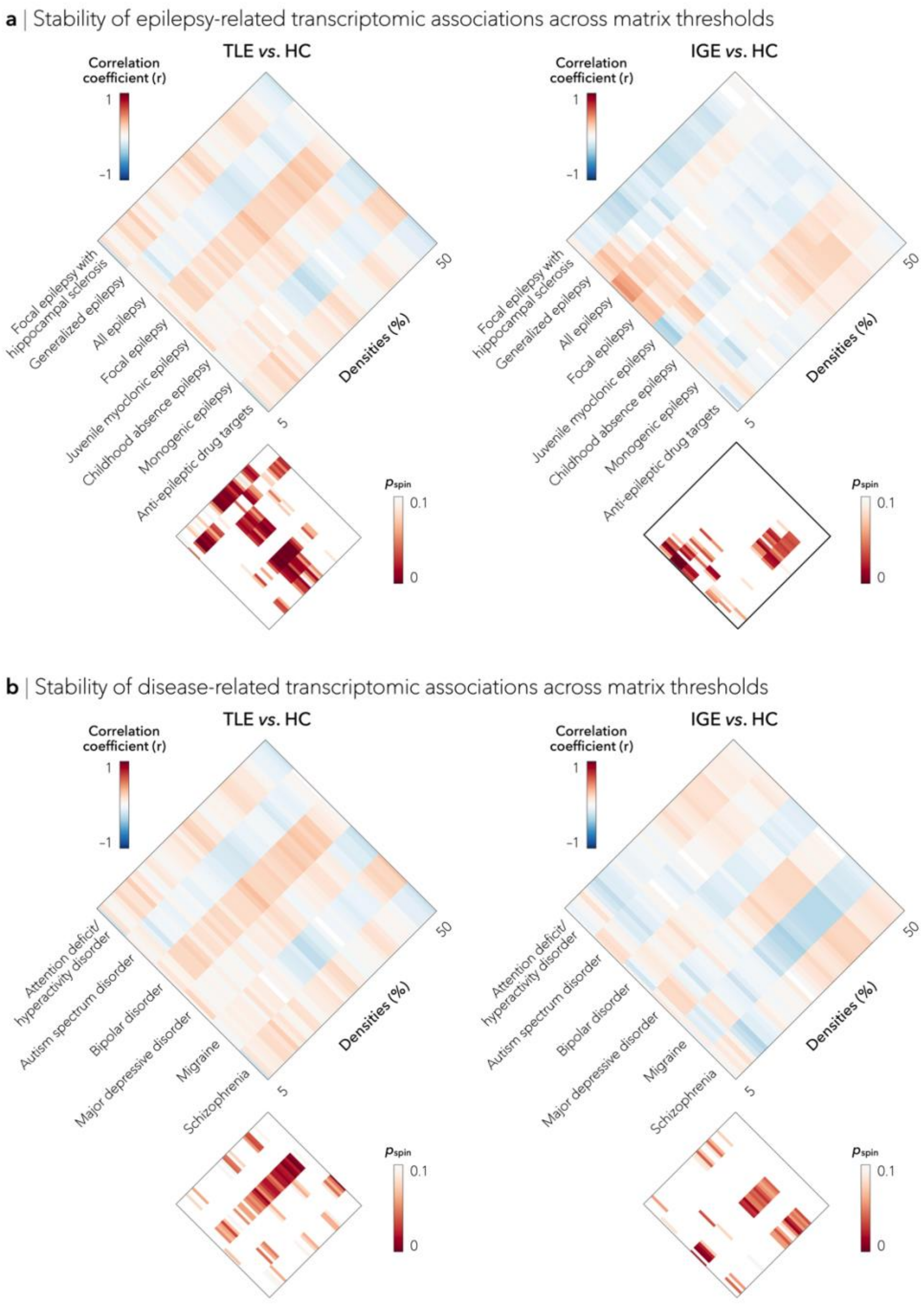

Supplementary Figure 12. **Stability of transcriptomic associations across matrix thresholds**. (**a**) Associations between epilepsy-related gene expression maps and multivariate findings across matrix thresholds. Statistical significance was assessed using one-tailed, non-parametric tests. (**b**) Associations between disease-related gene expression maps and multivariate findings across matrix thresholds. Statistical significance was assessed using one-tailed, non-parametric tests. *Abbreviations*: HC = healthy control, IGE = idiopathic generalized epilepsy, TLE = temporal lobe epilepsy, p_{spin} = *p*-value corrected against a null distribution of effects using a spatial permutation model.

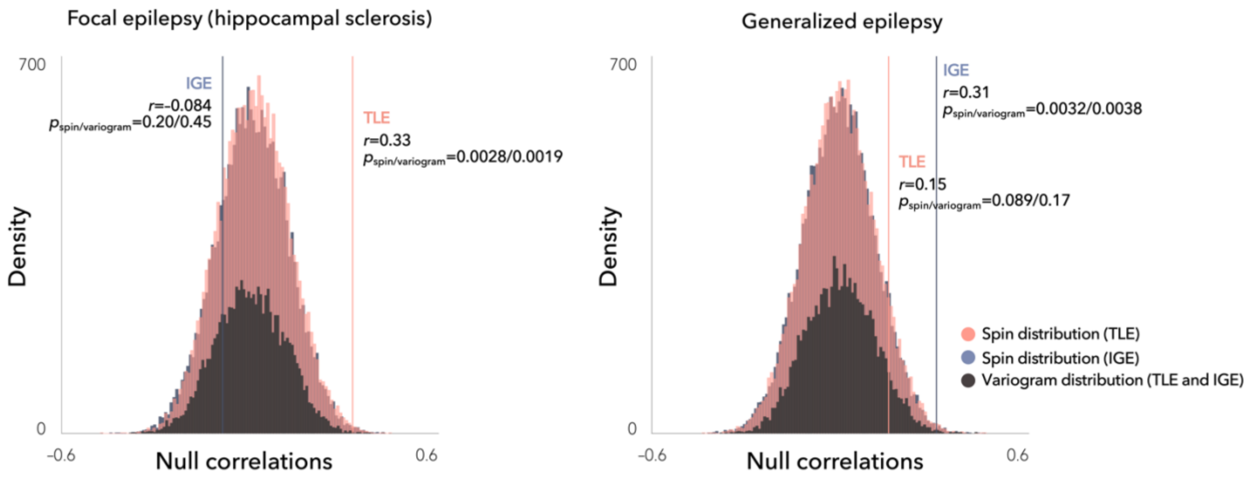

Supplementary Figure 13. **Cross-validation of cortical/subcortical spin permutation test**. Statistical significance of the imaging-transcriptomic spatial correlations from **Fig. 3B** was assessed using one-tailed spin permutation (pink and blue distributions) and variogram-matching (gray distribution) approaches. Both methods generated similar distributions and nearly identical *p*-values. *Abbreviations*: HC = healthy control, IGE = idiopathic generalized epilepsy, TLE = temporal lobe epilepsy, p_{spin} = *p*-value corrected against a null distribution of effects using a spatial permutation model, $p_{variogram}$ = *p*-value corrected against a null distribution of effects using a variogram matching model.

SUPPLEMENTARY REFERENCES

1. Consortium TILAE. Genome-wide mega-analysis identifies 16 loci and highlights diverse biological mechanisms in the common epilepsies. *Nature communications* **9**, (2018).
2. Santos R, *et al.* A comprehensive map of molecular drug targets. *Nature reviews Drug discovery* **16**, 19-34 (2017).
3. Allen AS, *et al.* Ultra-rare genetic variation in common epilepsies: a case-control sequencing study. *The Lancet Neurology* **16**, 135-143 (2017).
4. Demontis D, *et al.* Discovery of the first genome-wide significant risk loci for attention deficit/hyperactivity disorder. *Nature genetics* **51**, 63-75 (2019).
5. Grove J, *et al.* Identification of common genetic risk variants for autism spectrum disorder. *Nature genetics* **51**, 431-444 (2019).
6. Stahl EA, *et al.* Genome-wide association study identifies 30 loci associated with bipolar disorder. *Nature genetics* **51**, 793-803 (2019).
7. Howard DM, *et al.* Genome-wide meta-analysis of depression identifies 102 independent variants and highlights the importance of the prefrontal brain regions. *Nature neuroscience* **22**, 343-352 (2019).
8. Gormley P, *et al.* Meta-analysis of 375,000 individuals identifies 38 susceptibility loci for migraine. *Nature genetics* **48**, 856-866 (2016).
9. Pardiñas AF, *et al.* Common schizophrenia alleles are enriched in mutation-intolerant genes and in regions under strong background selection. *Nature genetics* **50**, 381-389 (2018).
