# Supplementary material for: Structural network alterations in focal and generalized epilepsy assessed in a worldwide ENIGMA study follow axes of epilepsy risk gene expression
Source: Nat Commun. 2022 Jul 27;13:4320. doi: 10.1038/s41467-022-31730-5 (PMC9329287; doi:10.1038/s41467-022-31730-5)
Supplement: Supplementary file 3 — Reporting Summary [file 41467_2022_31730_MOESM3_ESM.pdf]

nature portfolio

Corresponding author(s): Dr. Boris C Bernhardt

Last updated by author(s): Mar 18, 2022

Reporting Summary

Nature Portfolio wishes to improve the reproducibility of the work that we publish. This form provides structure for consistency and transparency in reporting. For further information on Nature Portfolio policies, see our Editorial Policies and the Editorial Policy Checklist.

Statistics

For all statistical analyses, confirm that the following items are present in the figure legend, table legend, main text, or Methods section.

n/a	Confirmed	
☐	☒	The exact sample size (n) for each experimental group/condition, given as a discrete number and unit of measurement
☐	☒	A statement on whether measurements were taken from distinct samples or whether the same sample was measured repeatedly
☐	☒	The statistical test(s) used AND whether they are one- or two-sided<br>*Only common tests should be described solely by name; describe more complex techniques in the Methods section.*
☐	☒	A description of all covariates tested
☐	☒	A description of any assumptions or corrections, such as tests of normality and adjustment for multiple comparisons
☐	☒	A full description of the statistical parameters including central tendency (e.g. means) or other basic estimates (e.g. regression coefficient) AND variation (e.g. standard deviation) or associated estimates of uncertainty (e.g. confidence intervals)
☐	☒	For null hypothesis testing, the test statistic (e.g. F, t, r) with confidence intervals, effect sizes, degrees of freedom and P value noted<br>*Give P values as exact values whenever suitable.*
☒	☐	For Bayesian analysis, information on the choice of priors and Markov chain Monte Carlo settings
☐	☒	For hierarchical and complex designs, identification of the appropriate level for tests and full reporting of outcomes
☐	☒	Estimates of effect sizes (e.g. Cohen's d, Pearson's r), indicating how they were calculated

Our web collection on statistics for biologists contains articles on many of the points above.

Software and code

Policy information about availability of computer code

Data collection	Derivative data (for example, summary statistics from published ENIGMA studies) and codes are openly accessible under the terms of the BSD-3-Clause license at http://github.com/ MICA-MNI/ENIGMA. Requests to work on a project with subject-level data can be proposed to the working group via the chairs (http://enigma.ini.usc.edu/). Gene expression data were obtained from the Allen Human Brain Atlas (https:// human.brain-map.org/) released by the Allen Institute for Brain Science. Cell type gene expression data were obtained from Zhu et al., 2018, Science.
Data analysis	Codes to analyze the data is openly available in the ENIGMA Toolbox v1.1.3 (BSD-3-Clause license; https://enigma-toolbox.readthedocs.io) and is complemented with expandable online documentation (http://enigma-toolbox.readthedocs.io). Models of cortical and subcortical surface morphology were generated with FreeSurfer 5.3.0 . Gene expression data were preprocessed using abagen v0.1.3 (https:// abagen.readthedocs.io). Variogram matching models were generated using BrainSpace v0.1.3 (https://brainspace.readthedocs.io/).

For manuscripts utilizing custom algorithms or software that are central to the research but not yet described in published literature, software must be made available to editors and reviewers. We strongly encourage code deposition in a community repository (e.g. GitHub). See the Nature Portfolio guidelines for submitting code & software for further information.

March 2021

Data

Policy information about availability of data

All manuscripts must include a data availability statement. This statement should provide the following information, where applicable:

- Accession codes, unique identifiers, or web links for publicly available datasets
- A description of any restrictions on data availability
- For clinical datasets or third party data, please ensure that the statement adheres to our policy

All data needed to evaluate the conclusions in the paper are present in the paper and in the references cited. Additional data related to this paper are freely available for download (https://github.com/MICA-MNI/ENIGMA).

Field-specific reporting

Please select the one below that is the best fit for your research. If you are not sure, read the appropriate sections before making your selection.

☒ Life sciences ☐ Behavioural & social sciences ☐ Ecological, evolutionary & environmental sciences

For a reference copy of the document with all sections, see nature.com/documents/nr-reporting-summary-flat.pdf

Life sciences study design

All studies must disclose on these points even when the disclosure is negative.

Sample size	We studied 866 adult epilepsy patients and 1,328 healthy controls from 18 centres in the international Epilepsy Working Group of ENIGMA 39. No sample size calculation was needed as we used all available individuals from the largest epilepsy neuroimaging dataset to date. For neuroimaging analysis, a sample of >80 can already have sufficient power (https://doi.org/10.1002/hbm.25217); sample sizes used in this study are much higher (all groups >280).
Data exclusions	Data exclusion criteria were pre-established from the original ENIGMA-Epilepsy paper (Whelan et al., 2018, Brain). We excluded participants with a progressive or neurodegenerative disease (e.g., Rasmussen's encephalitis, progressive myoclonus epilepsy), malformations of cortical development, tumors, or prior neurosurgery. The rationale was to ensure that disease effects observed in patients were caused by epilepsy (either TLE or IGE) and not by progressive or neurodegenerative diseases.
Replication	We conducted several sensitivity analyses: (i) global and regional structural covariance differences were assessed in each site independently, (ii) multivariate topological findings and associations between network-level findings and gene expression maps were assessed across the range of possible matrix thresholds, and (iii) imaging-transcriptomic associations were assessed in left and right TLE independently. The results of our analyses were highly consistent, suggesting that our findings were not affected by differences in scanners or sites or methodological choices.
Randomization	Allocation of participants was not random. Epilepsy specialists at each center diagnosed patients according to the seizure and syndrome classifications of the ILAE. Inclusion of adults with TLE was based on the combination of electroclinical features and MRI findings typically associated with underlying hippocampal sclerosis. Inclusion of adults with IGE was based on the presence of tonic-clonic, absence, or myoclonic seizures with generalized spike-wave discharges on EEG. Healthy controls had no history of mental disorders and were statistically matched for age and sex to the epilepsy subgroups at each site.<br><br>We controlled for several covariates, as follows:<br>- Site and scanner effects were mitigated using CovBat, a post-acquisition statistical batch normalization process used to harmonize between-site and between-protocol effects in mean, variance, and covariance, while protecting biological covariates (e.g., disease status).<br>- Cortical thickness and volumetric measures were statistically corrected for age and sex.<br>-Residualized data were z-scored relative to site-matched pooled controls and sorted into measures that were ipsilateral/contralateral to the focus.
Blinding	Blinding was not relevant to our study as we did not collect new data (all data came from previously published sources). All participant imaging data were deidentified and were assigned a site-specific ID.

Reporting for specific materials, systems and methods

We require information from authors about some types of materials, experimental systems and methods used in many studies. Here, indicate whether each material, system or method listed is relevant to your study. If you are not sure if a list item applies to your research, read the appropriate section before selecting a response.

Materials & experimental systems

n/a	Involved in the study	
☒	☐	Antibodies
☒	☐	Eukaryotic cell lines
☒	☐	Palaeontology and archaeology
☒	☐	Animals and other organisms
☐	☒	Human research participants
☒	☐	Clinical data
☒	☐	Dual use research of concern

Methods

n/a	Involved in the study	
☒	☐	ChIP-seq
☒	☐	Flow cytometry
☐	☒	MRI-based neuroimaging

Human research participants

Policy information about studies involving human research participants

Population characteristics	We studied 866 adult epilepsy patients (377 males, mean age±SD=33.82±9.48 years) and 1,328 healthy controls (588 males, mean age±SD=30.74±8.30 years) from 18 centres in the international Epilepsy Working Group of ENIGMA. Our analyses focused on two patient subcohorts with site-matched healthy controls: TLE with neuroradiological evidence of hippocampal sclerosis (nHC/TLE=1,083/578, 257 right-sided focus) and IGE (nHC/IGE=911/288).
Recruitment	Recruitment includes patients with epilepsy and age- and sex-matched healthy controls from 18 centres around the world. We restricted our analysis to adults aged 18-50 years to limit potential bias related to the development of a wide range of diseases and conditions in early (i.e. childhood and early adolescence) and mid- to late adulthood. Neuroimaging measures were nevertheless corrected for age and sex to limit potential bias.<br><br>Moreover, epilepsy specialists at each center diagnosed patients according to the seizure and syndrome classifications of the ILAE. The International League Against Epilepsy (ILAE) Classification framework is the gold standard tool for the diagnosis of individuals presenting with seizures. Specifically, inclusion of adults with TLE was based on the combination of electroclinical features and MRI findings typically associated with underlying hippocampal sclerosis. Inclusion of adults with IGE was based on the presence of tonic-clonic, absence, or myoclonic seizures with generalized spike-wave discharges on EEG.
Ethics oversight	Local institutional review boards and ethics committees approved each included cohort study, and written informed consent was provided according to local requirements. Details of research ethics committee approval for each site is provided in Supplementary Table 3. Briefly: Bern (Ethics commission of the Canton of Bern), Bonn (Medical Ethics Committee of the University of Bonn), Brussels (Ethics Commission of Erasme Hospital), CUBRIC (Southmead Research Ethics Committee), EPICZ (Research Ethics Committee of University “Magna Graecia”), EPIGEN_3.0 (SJH/TUH Joint Research Ethics Committee), Greifswald (University Medicine Greifswald's Ethics Committee), IDIBAPS-HCP (The Ethics Committee of Hospital Clinic de Barcelona), KCL_CNS (KCL College Research Ethics Committees), Kuopio (The Research Ethics Committee of the Northern Savo Hospital), MNI (The Ethics Committee of the Montreal Neurological Institute and Hospital), NYU (New York University's Institutional Review Board), RMH (The Royal Melbourne Hospital Human Research Ethics Committee), UCSD (The institutional review board at UC San Diego), UNAM (The Ethics Committee of the Neurobiology Institute of the Universidad Nacional Autónoma de México), UNICAMP (Comitê de Ética em Pesquisa da Universidade Estadual de Campinas), UNIMORE (The human ethics committee of the University of Modena and Reggio Emilia), XMU (Xinjiang Medical University (XMU) Research Ethics Committee).

Note that full information on the approval of the study protocol must also be provided in the manuscript.

Magnetic resonance imaging

Experimental design

Design type	Structural T1w MRI
Design specifications	one run per subject
Behavioral performance measures	n/a: Behavioral data were not available as participants did not perform any tasks.

Acquisition

Imaging type(s)	Structural T1w MRI
Field strength	3T
Sequence & imaging parameters	All participants underwent structural T1-weighted brain MRI scans at each of the 18 participating centers, with scanner descriptions and acquisition protocols detailed elsewhere (Whelan et al., 2018, Brain).
Area of acquisition	Whole-brain

Diffusion MRI ☐ Used ☒ Not used

Preprocessing

Preprocessing software	Images were independently processed by each center using the standard ENIGMA workflow. In brief, models of cortical and subcortical surface morphology were generated with FreeSurfer 5.3.0 99. Based on the Desikan-Killiany anatomical atlas, cortical thickness was measured across 68 grey matter brain regions and volumetric measures were obtained from 12 subcortical grey matter regions (bilateral amygdala, caudate, nucleus accumbens, pallidum, putamen, thalamus) as well as bilateral hippocampus.
Normalization	Cortical thickness and volumetric measures were corrected for age and sex. Residualized data were z-scored relative to site-matched pooled controls and sorted into measures that were ipsilateral/contralateral to the focus.
Normalization template	Data were mapped to a common surface-based parcellation (Desikan-Killiany anatomical atlas).
Noise and artifact removal	Data were harmonized across scanners and sites using CovBat—a batch-effect correction tool that uses a Bayesian framework to improve the stability of the parameter estimates.
Volume censoring	n/a: Volume censoring was not performed as this study only analyzed structural T1w images.

Statistical modeling & inference

Model type and settings	Surface-based linear models and Cohen's d effect sizes compared morphological measures in patients relative to controls.
Effect(s) tested	NO ANOVA or factorial designs were included in our study.

Specify type of analysis: ☒ Whole brain ☐ ROI-based ☐ Both

Statistic type for inference (See Eklund et al. 2016)	Parcel-wise.
Correction	Findings were corrected for multiple comparisons using the false discovery rate (FDR) procedure

Models & analysis

n/a	Involved in the study
☒	☐ Functional and/or effective connectivity
☐	☒ Graph analysis
☐	☒ Multivariate modeling or predictive analysis

Graph analysis	From the weighted, thresholded structural covariance networks, we computed three global metrics using standard formulas: (i) mean clustering coefficient, which quantifies the tendency for brain regions to be locally interconnected with neighboring regions, (ii) mean path length, which quantifies the mean minimum number of edges (i.e., connection between two regions) that separate any two regions in the network, and (iii) small-world index (mean clustering coefficient divided by mean path length), which quantifies both local and global properties. These two metrics, along with their combination (i.e., small-world index), are the most widely used graph theoretical parameters to describe the topology of complex networks. Each measure was normalized relative to corresponding measures from 1,000 randomly generated networks with similar degree and weight properties, and subsequently averaged across all cortical and subcortical regions, separately.
Multivariate modeling and predictive analysis	To signify an overall load of anomalies, we subsequently compared the aggregate of clustering coefficient and path length differences in patients relative to controls using multivariate surface-based linear models.
